# Health Tract, No. 284

**Published:** 1867-03

**Authors:** 


HEALTH TRACT. No. 284.

### ARMY DISEASE AND DEATH.

There was enlisted during the great Rebellion, on the Union side, — 2,688,523

| | |
|---|---|
| Regulars already in the army, - - - - - - - | 14,000 |
| Total, - - - - - - - - - - - - - | 2,702,523 |
| The number who left the army alive was - - - - - | 2,408,103 |
| So there died of battle and disease - - - - . - | 280,420 |
| The total number of colored troops was - - - - - | 180,005 |

The total number of deaths among the whites was one out of every ten; while one out of every six of the colored troops perished.

Out of every eight deaths among the whites, three died on the field and five from disease. Of every nine deaths among the colored, one died on the field and eight from disease; this shows that greater intelligence enables a man to endure more, and that this intelligence tends to exempt from disease. Many cases are recorded where captains of companies and commanders of vessels have endured the sufferings and exposures under which the common men perished. On one occasion the superior intelligence and moral courage of Dr. Kane, a small and feeble man, by sustaining the spirits of the crew, saved them from perishing with cold; for the same reason, an intelligent man will perform more labor under given circumstances than an uneducated workman. Hence an educated mechanic will always do his work better, faster and easier than the uneducated;—thus, whatever pursuit a child may be intended to follow, a good education is a very important element of success.

Not only is intelligence an element of health in the pursuits and occupations of life, but experience is another; the regular soldiers in the Union Army had a mortality of fifteen per cent. less than the volunteers; it was observed in all parts of the country, that in the commencement of a march the volunteer would encumber himself with this, that and the other supposed indispensable article, amounting in all to half as much again in weight as would the experienced soldier; but after the first few miles, first one thing would be thrown aside, then another, until at the close of the very first day's travel, the roadway of the line of march would be fairly covered with every variety of articles of supposed comfort that a person could think of, and many a man who could in the morning have set up a young hotel or clothing store, would at the close of the second or third day's march find that his entire stock in trade amounted to a tin cup and a blanket. Two important facts stand out in bold relief in these army returns: First, two died of disease where one died in battle. Second, Two colored troops were lost to one of white, in proportion. Third—Intelligence is a great help to a man in bearing the difficulties and exposures of life, enabling him to survive hardships under which the uncultivated soon sink into the grave.

